# Effects of thymectomy on late-onset non-thymomatous myasthenia gravis: systematic review and meta-analysis

**DOI:** 10.1186/s13023-021-01860-y

**Published:** 2021-05-20

**Authors:** Jinwei Zhang, Yuan Chen, Hui Zhang, Zhaoyu Yang, Peng Zhang

**Affiliations:** Department of Cardiothoracic Surgery, Tianjin Medical University General Hospital, No.154, Anshan Road, Tianjin, China

**Keywords:** Myasthenia gravis, Early onset, Late onset, Thymectomy, Remission, Systematic review

## Abstract

**Background:**

The effects of thymectomy on late-onset non-thymomatous myasthenia gravis (NTMG) remain controversial. The objective of this study was to conduct a systematic review in order to answer two questions pertinent to late-onset NTMG: (1) do patients with late-onset NTMG experience the same effects from thymectomy as their early-onset counterparts? (2) Compared with conservative treatment, does thymectomy have any benefits for late-onset NTMG patients?

**Methods:**

We searched the PubMed, EMBASE, and Cochrane Library databases for studies published from January 1, 1950 to March 10, 2021. Outcomes were measured via clinical stable remission/pharmacological remission (CSR/PR) and improvement rates. We used Stata software to analyze the data.

**Results:**

We ultimately included a total of 12 observational articles representing the best evidence answering the questions of our study objective. Of these, nine studies, which included 896 patients overall (766 early-onset and 230 late-onset), compared postoperative outcomes between early- and late-onset NTMG. The remaining three articles, which included 216 patients (75 in the thymectomy group and 141 in the conservative-treatment group), compared thymectomy with conservative treatment for late-onset NTMG. The early- versus late-onset NTMG studies demonstrated that patients in the former category were 1.95 likelier than their late-onset counterparts to achieve clinical remission (odds ratio [OR] 1.95; 95% confidence interval [CI] 1.392.73; *I*^2^=0%). No difference was seen in improvement or remission+improvement rates between these two groups. When comparing thymectomy with conservative treatments in late-onset NTMG patients, neither did we observe any difference in CSR/PR.

**Conclusion:**

We found that late-onset NTMG patients had a lower chance of achieving CSR after thymectomy than early-onset patients. Thymectomy in late-onset NTMG also yielded no benefit to CSR or PR compared with conservative treatments. In late-onset NTMG patients, thymectomy should therefore be performed with caution, and the appropriate cutoff between early- and late-onset MG should be further explored in order to tailor and execute the proper therapeutic strategies.

## Background

Myasthenia gravis (MG) is an autoimmune disease characterized by fatigable weakness of cranial and skeletal muscles with elevated titers of acetylcholine receptor (AChR), muscle-specific receptor tyrosine kinase (MuSK) autoantibodies, or other AChR-related proteins that affect the postsynaptic membrane at the neuromuscular junction [1]. The incidence rate of MG varies with age, gender, ethnicity, thymic histology, clinical presentation, and muscular autoantibodies [2]. The estimated range of incidence is 0.32.8 per 100,000 worldwide, and the median global estimated prevalence is 10 per 100,000 [3]. In China, analysis across age groups shows that the incidence of MG increases steadily with age over the first seven decades of life, peaking in the 7074years age group (1.89 per 100,000) in adults [4].

According to the age at which the first symptom manifests, MG can be divided into early-onset and late-onset subgroups. The cutoff age is usually recommended as 4050years, less often 6065years [5, 6]. The two groups can differ in sex ratio, thymic histology, autoantibody titers, and reaction to thymectomy [7, 8]. Thymectomy in non-thymomatous MG (NTMG) patients has mostly been conducted in early-onset patients. Current guidelines and consensus statements for patients with early-onset NTMG note that these patients most often have thymic hyperplasia and advise that removal of the hyperplastic thymus might contribute to decreased antibody production [8, 9].

Some controversy surrounds the effect of thymectomy in late-onset NTMG patients. The landmark MGTX trial [10] was a multicenter, randomized, rater-blinded study that compared the effects of thymectomy+prednisone with those of prednisone alone in NTMG patients with generalized AChR-MG over a 3-year period. Patients who underwent thymectomy had a lower time-weighted average Quantitative Myasthenia Gravis (QMG) score and time-weighted average alternate-day prednisone dose. In addition, the results favored thymectomy for patients who took azathioprine or were hospitalized for MG exacerbations. However, the MGTX trial did not definitively establish the role of thymectomy in late-onset NTMG, nor did it find a significant difference in prednisone dosage or QMG score between the two treatment groups in patients age50years [10]. Similar results have been observed in other studies [11, 12], though the results thereof have suggested a potential benefit of thymectomy in late-onset NTMG [13,15]. Given the contradictory evidence on the effects of thymectomy, we conducted this systematic review and meta-analysis with the aim of answering two questions pertaining to late-onset NTMG: (1)Compared with early-onset NTMG patients, do late-onset patients experience any effects from thymectomy? (2) Compared with standard conservative treatment, does thymectomy have any benefits in late-onset NTMG patients?

## Materials and methods

### Data sources

We searched the electronic databases PubMed, EMBASE, and the Cochrane Library for studies published from January1, 1950 to March 10, 2021. The MeSH terms, EMTREE terms, and keywords used were myasthenia gravis AND thymectomy AND remission or improved or improvement AND aged or elder or older or elderly or late-onset or non-thymomatous. With no restrictions on language or publication status. For non-English articles needing a full-text review, we used translation software to perform detailed evaluations. Every publication, regardless of article type, was carefully screened for eligibility. We then further screened the references of relevant publications that we found in our search for eligible studies.

### Study selection

Two independent reviewers assessed article eligibility; disagreements were resolved through subsequent discussion. Studies were included if they met the following inclusion criteria. (1) The study cohort consisted of NTMG patients who received thymectomy, regardless of surgical method. (2) Outcome comparisons were measured by remission and/or improvement. Remission was defined as complete stable remission (CSR) or pharmacological remission (PR) according to the criteria of the Myasthenia Gravis Foundation of America (MGFA) [16]. Studies using similar definitions of remission were also included; in these cases, we compared these definitions to decrease selection bias. (3) Comparison data could be extracted from the study; i.e., data on thymectomy versus conservative treatment (anticholinesterase, corticosteroids, or immunosuppressants administered either alone or in combination) in late-onset NTMG patients, or early-onset versus late-onset NTMG patients after thymectomy. (4) The cutoff age range was 4060years.

Studies were excluded if they met the following criteria: (1) Children/juvenile MG only; (2) ocular MG only; or (3) refractory MG or MG crisis only.

### Data extraction and quality assessment

Two authors independently extracted data from eligible studies and summarized them using a data extraction form. For randomized controlled trials (RCTs), methodological quality was assessed using the quantitative five-point Jadad scale [17]; quality was considered high if Jadad score>3. In addition, we used the NewcastleOttawa Scale (NOS) to assess observational studies [18]. We considered scores of7 points to indicate high methodological quality.

### Data synthesis

Data analyses were performed using Stata statistical software version 15 (StataCorp., College Station, TX, USA). If *I*^2^>0, we applied a random-effects model. Substantial heterogeneity was considered present when *I*^2^>75%; in these cases, the combination was considered inappropriate, and the results were presented in narrative form. We also assessed the probability of publication bias using Peters regression test [19]. Statistical significance was defined as two-tailed *P*<0.05.

## Results

### Search results and study characteristics

**Fig. 1:**
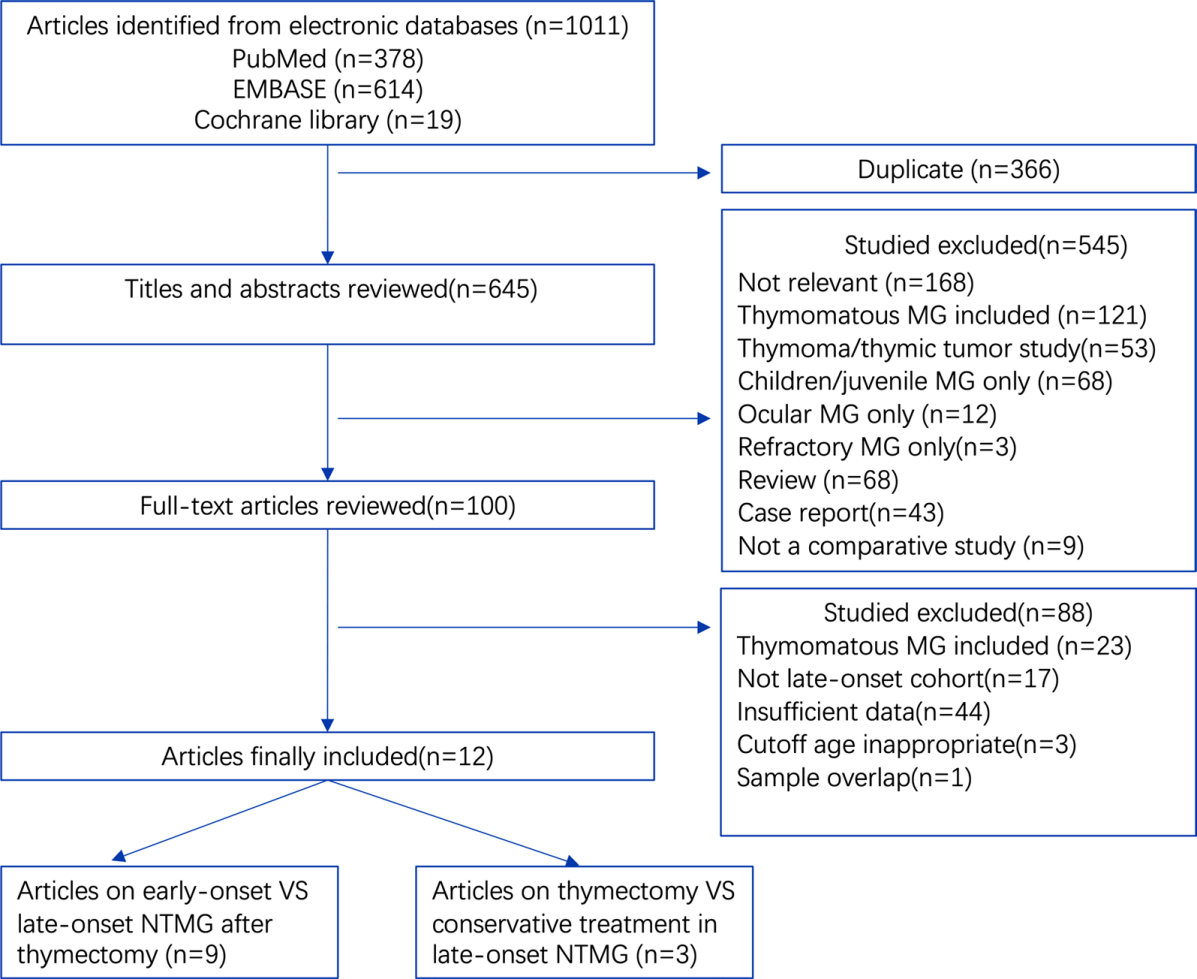
Flow diagram showing the study selection procedure

In our electronic data search we identified 378 articles in PubMed, 614 in EMBASE, and 19 in the Cochrane Library. After excluding duplicates and analyzing titles and abstracts, we selected 100 articles, of which we obtained full paper copies. From these, we ultimately selected a final 12 publications. Nine studies compared outcomes between early- and late-onset NTMG patients after thymectomy [12, 20,27], while three compared the effects of thymectomy with those of conservative treatment on late-onset NTMG [11, 13, 15] (Fig.1). All studies were observational. The main characteristics and quality assessments of the selected studies are summarized in Tables 1 and2.

**Table 1:** Demographic data of studies comparing early-onset with late-onset NTMG after thymectomy

Author/year/country	Study design	Study period	Follow-up (y) mean/range	Age (y) (cutoff/range)	Early-onset (events/all)		Late-onset (events/all)		Thymic histology hyperplasia/involution/normal	Anti-AChR-ab (+//ND)	Preoperative classification	Surgical procedures	Medical treatment	NOS score
					CSR	Improved	CSR	Improved						
Liu/2015/China [20]	Single-center retrospective	20072011	5.2/3.17.2	40/NA	27/57	NA	21/46	NA	68/35/0	54/21/28	I 25/IIa 25/IIb 15/IIIa 17/IIIb 18/IV 3 MGFA	Bilateral/Right VATS	Anticholinesterase; corticosteroid; Ig	8
Lin/2010/Taiwan [12]	Single-center retrospective	19952004	3.6/111	40/578	16/42	NA	4/18	NA	42/14/4	39/10/11	I 22/II 30/III 4/IV 1/V 3 MGFA	Right-VATS/TS	anticholinesterase; corticosteroid	8
Zieliski/2004/Poland [21]	Single-center retrospective	19961999	NA/3.56.5	40/1470	25/52	NA	2/6	NA	33/15/10	NA	I 5/IIa 19/IIb 34 Osserman	TS	Anticholinesterase; corticosteroid; immuno-suppressant	8
Mantegazza/2003/Italy [22]	Single-center prospective	NA	NA 16	40/NA	72/185	NA	2/21	NA	130/76/0	169/37/0	I 19/IIIa 63/IIIb 99/IVb 25 MGFA	Bilateral VATET/TS	Anticholinesterase; immuno-suppressant	8
Mack/1996/USA [23]	Multicenter retrospective	19921995	NA/0.33.9	40/984	5/21	14/21	1/6	3/6	19/2/6	NA	I 2/II 23/III 2 Osserman	VATS	Anticholinesterase; steroids	7
Frist/1994/USA [24]	Single-center retrospective	19711992	NA/0.821	45/267	12/33	19/33	2/9	3/9	NA	20/8/14	I 2/II 7/III 11/IV 19/V 3 Oosterhuis	TS	Anticholinesterase; corticosteroid	7
Maggi/1989/Italy [25]	Single-center retrospective	19731987	NA/510	40/NA	137/326	152/326	31/117	67/117	NA	NA	I 27/IIa 256/IIb 200/III 17 own classification	TC/TC+TS	Anticholinesterase; corticosteroid; immuno- suppressant; plasmapheresis	8
Monden/1985/Japan [26]	Single-center retrospective	NA	5/NA	50/1659	21/32	9/32	2/4	2/4	NA	NA	I 5/IIa 29/IIb 67/III 1 Oosterhuis	TS	NA	8
Rubin/1981/USA [27]	Single-center retrospective	19611982	NA/0.515	40/954	9/18	9/18	1/3	2/3	13/3/5	6/15/0	II 6/III 6/IV 8/V 1 Osserman	TS	Anticholinesterase; corticosteroid; plasmapheresis	7

*NTMG* non-thymomatous myasthenia gravis, *AntiAChR-ab* anti-acetylcholine receptor antibody, *CSR* complete stable remission, *TS* trans-sternal thymectomy, *TC* transcervical thymectomy, *VATS* video-assisted thoracoscopic surgery, *MGFA* Myasthenia Gravis Foundation of America, *NOS* NewcastleOttawa scale, *NA* not available, *ND* not determined, *IG* immunoglobulin

#### Descriptive analysis of studies

##### Studies comparing outcomes of early-onset versuslate-onset NTMG patients after thymectomy (n=9)

1. Liu et al*.* [20], at a single center in China, retrospectively reviewed 103 consecutive patients who received thymectomy for NTMG. Median duration of follow-up was 5.2years. Overall, 48 patients achieved CSR, and 1-, 2-, 3-, 4-, 5-, and 6-year CSR rates were 9%, 25%, 40%, 45%, 52%, and 56%, respectively. Univariate analysis of age did not correlate significantly with CSR. With the cutoff age at 40years, 47% (27/57) of early-onset and 46% (21/46) of late-onset patients achieved CSR 5years after surgery. CSR was defined by the MGFA standard.
2. Lin et al*.* [12], at a single center in Taiwan, retrospectively studied postoperative outcomes of 60 NTMG patients, following up with them for a mean of 44months. Age<40years was one of the three prognostic factors associated with better remission rate. Patients achieving CSR were 38% (16/42) in the<40years onset age group, compared with 22% (4/18) in the>40years onset age group. CSR was defined by the Clinical Research Standards of the MGFA.
3. Zieliski et al*.* [21], in a single-center retrospective study in Poland, compared the late results of basic and extended trans-sternal thymectomies. Mean follow-up was 6years in the basic-thymectomy group and 4years in the extended-thymectomy group. The complete-remission (CR) rate in the extended group was statistically higher than that in the basic group. However, age (<40 or>40years) had no effect on CR or rates of negative results in either group. Considering that extended thymectomy is already widely recommended as standard procedure [28], we extracted age data from the extended group. We found that 48.1% (25/52) of patients in the early-onset subgroup and 33.3% (2/6) of those in the late-onset subgroup achieved CSR. CR was defined as no symptoms of MG and no need for antimyasthenic medication.
4. Mantegazza et al*.* [22], in a prospective nonrandomized single-center study in Italy, evaluated 206 NTMG patients for 16years after video-assisted thoracoscopic extended thymectomy (VATET) or trans-sternal thymectomy. Age<40years at onset, thymic hyperplasia, and anticholinesterase drug usage were associated with a significantly greater probability of achieving CSR. In the early-onset group, 38.9% (72/185) of patients achieved CSR, while 9.5% (2/21) did so in the late-onset group. CSR was defined by the MGFA standard.
5. Mack et al*.* [23], in a multicenter retrospective study in the USA, included 27 NTMG patients who had undergone thymectomies at four institutions by video-assisted thoracoscopic surgery (VATS) between 1992 and 1995, with 40years as the cutoff age. CR, which was defined as no symptoms and no need for medications, occurred in 5 of 21patients in the early-onset group versus 1 of 6 in the late-onset group. Follow-up duration for all patients who achieved CR exceeded 1year, which was in accordance with the MGFA CSR standard, although overall follow-up time was relatively short (447months).
6. Frist et al*.* [24], at a single center in Tennessee, USA, retrospectively reviewed the clinical courses of 42 NTMG patients treated with thymectomy via median sternotomy, with a follow-up duration of 10months21years. Twelve (36.4%) patients in younger group and two (22.2%) in the older group achieved remission; improvement was seen in 19 (57.6%) and 3 (33.3%) patients from these groups, respectively. Outcome criteria were graded according to change from preoperative status to time of most recent follow-up, in accordance with Oosterhuis classification [29]: remission implied no symptoms and no medication, much improved was defined as a decrease of2 stages, and improved was defined as a decrease of 1 stage.
7. Maggi et al*.* [25], at a single center in Italy, retrospectively studied the post-operative outcomes of 500 NTMG patients. Follow-up data were obtained in 443 cases. CR was achieved in 42% (137/326) of patients<40years of age but in only 26.4% (31/117) of patients in the older group, a statistically significant difference. The improvement rate was 46.6% (152/326) and 57.2% (67/117), respectively, in these groups. Remission was defined as total disappearance of symptoms with no need for treatment; improvement was defined as total disappearance of symptoms with occasional need for drugs, or mild symptoms with modest drug intake.
8. Monden et al*.* [26], in a retrospective single-center study in Japan, compared the clinical features and effects of thymectomy between elderly and young NTMG patients with a cutoff age of 50years. Rates of remission and palliation (remission+improvement) after thymectomy in elderly patients were respectively 0 and 66.7% at 6months, 0 and 50% at 1year, 50% and 100% at 3years, and 50% and 100% at 5years. The respective rates in young patients were 18.6% and 88.4% at 6months, 28.2% and 93.6% at 1year, 50% and 98.1% at 3years, and 65.6% and 93.8% at 5years. The difference between the two groups was not significant. Evaluation criteria were defined as follows: remission=no symptoms, or minimal residual symptoms without medication, plus a complete return to work; improved=increased activity with the same amount of medication or less.
9. Rubin et al*.* [27], in a single-center retrospective study in the USA, reviewed 21 NTMG patients treated with thymectomy with up to 15years of follow-up. Response to thymectomy was graded as follows: CR=without medication for90days; improvement=increased activity with the same or decreased amount of medication. With 40years as the cutoff age, 50% of patients (9/18) achieved CR in the early-onset group, and the other half achieved improvement. For late-onset patients, CSR and improvement rates were 33.3% (1/3) and 66.7% (2/3), respectively.

##### Studies comparing the effects of thymectomy versus conservative treatment for late-onset NTMG (n=3)

1. Kim et al*.* [15], in a retrospective cohort study in South Korea, compared MGFA post-intervention status (MGFA-PIS) in patients with AChR antibodypositive (AChR-ab^+^) generalized MG who underwent thymectomy at50years of age *versus* those who received medical treatment only. Follow-up duration was 30132months. Cumulative incidence of PR and CSR was significantly higher in the thymectomy group than in the medical-treatment group. Of the 34 patients in the thymectomy group, 6 achieved CSR, and 18 achieved PR. In the medical-treatment group, the success rates were 20/105 for CSR and 28/105 for PR. Note that the number of patients in the medical-treatment group who achieved CSR was not directly shown in the original article; we obtained this data by creating KaplanMeier plots between the two groups using Engauge Digitizer software version 12.2.1.
2. Kawaguchi et al*.* [13], in a multicenter retrospective study in Japan, performed thymectomy on 20 out of 34 patients (59%) with late-onset (age>50years) NTMG. Mean follow-up duration was 9.6years. Six (30%) patients in the thymectomized group and three (21%) in the non-thymectomized group achieved remission according to the MGFA-PIS standard (no symptoms; MGFA, score 0). The between-group difference was not statistically significant.
3. Romi et al*.* [11], in a retrospective single-center study in Norway, evaluated whether the presence of titin and/or RyR antibodies interfered with thymectomy outcome in NTMG patients. Follow-up duration was 25years. Four (titin and RyR antibody negative) thymectomized patients achieved full CR, while two achieved PR (immunosuppressive drugs were used). CR was seen in three non-thymectomized patients (titin antibody positive); four patients achieved PR. Clinical MG remission was defined as the disappearance of all myasthenic symptoms with (PR) or without (CSR) ongoing medication treatment.

### Effects of thymectomy in early-onset versus late-onset patients with NTMG (meta-analysis)

**Fig. 2:**
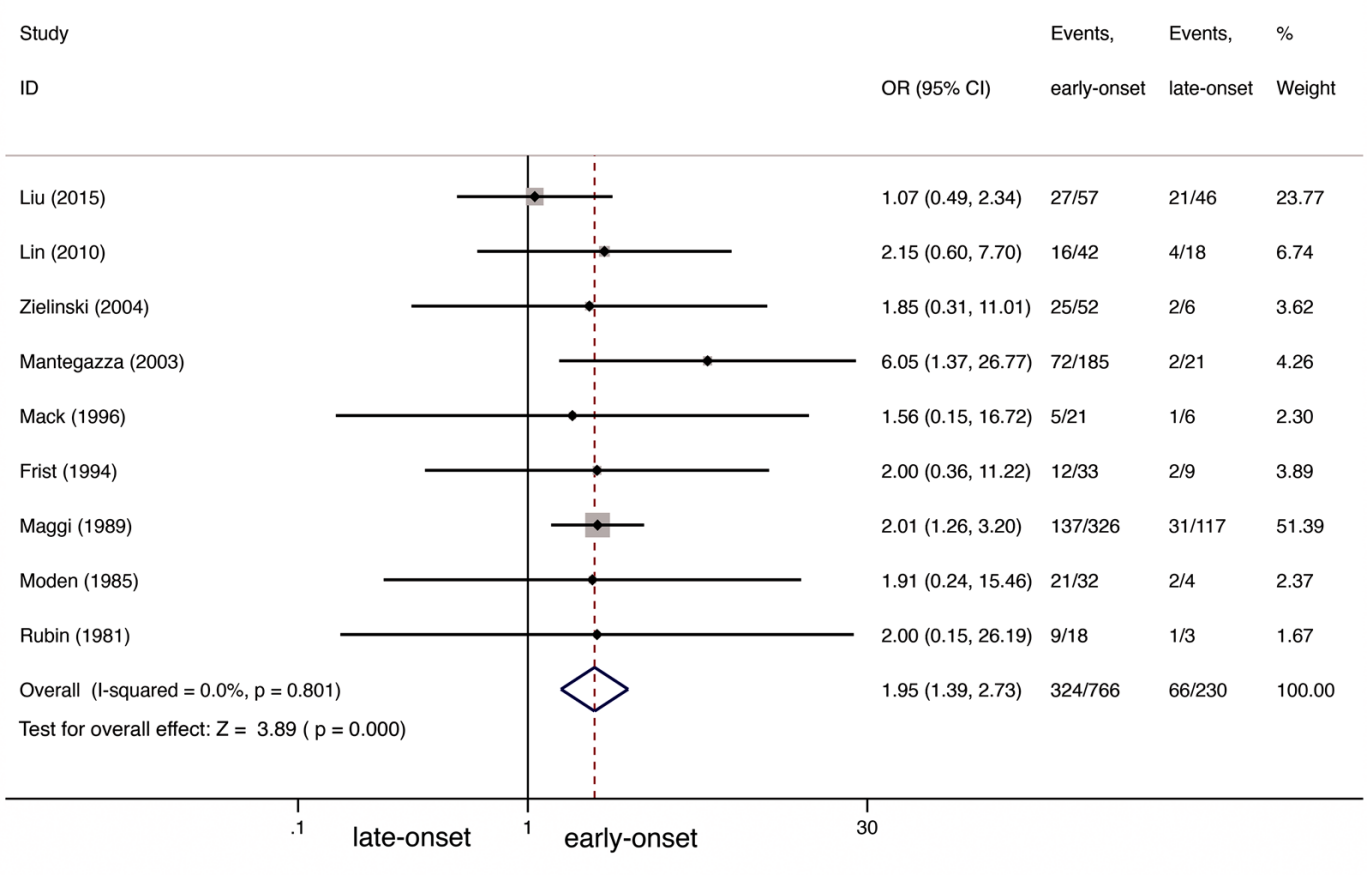
CSR comparison between early-onset and late-onset NTMG after thymectomy

The authors of all nine studies analyzed CSR between their early- and late-onset NTMG participants [12, 20,27], which in total included 766 patients in early-onset groups and 230 in late-onset groups. We observed a difference between the two (odds ratio [OR] 1.95; 95% confidence interval [CI] 1.392.73; *I*^2^=0%; Fig.2): the early-onset groups had 1.95 greater success in achieving CSR than the late-onset groups. No evidence of publication bias was detected by Peters test (*P*=0.571).

**Fig. 3:**
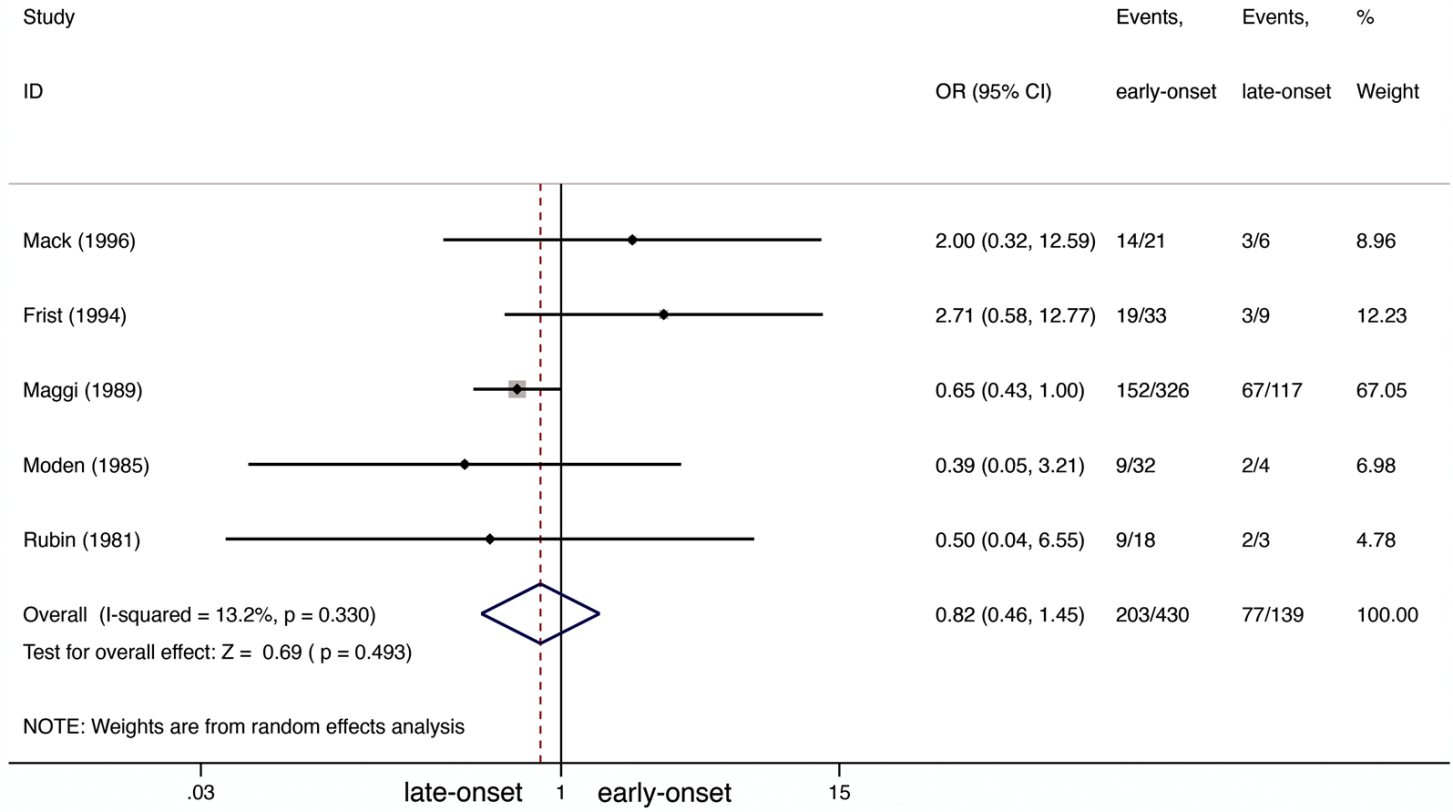
Improvement comparison between early-onset and late-onset NTMG after thymectomy

Only five studies included data on improvement after thymectomy in early-onset *versus* late-onset NTMG [23,27], including a total of 430 early-onset and 139 late-onset patients. As shown in Fig.3, we saw no between-group difference in improvement rate (OR 0.82; 95% CI 0.461.45; *I*^2^=13.2%; Fig.3). Peters test detected no evidence of publication bias (*P*=0.544).

**Fig. 4:**
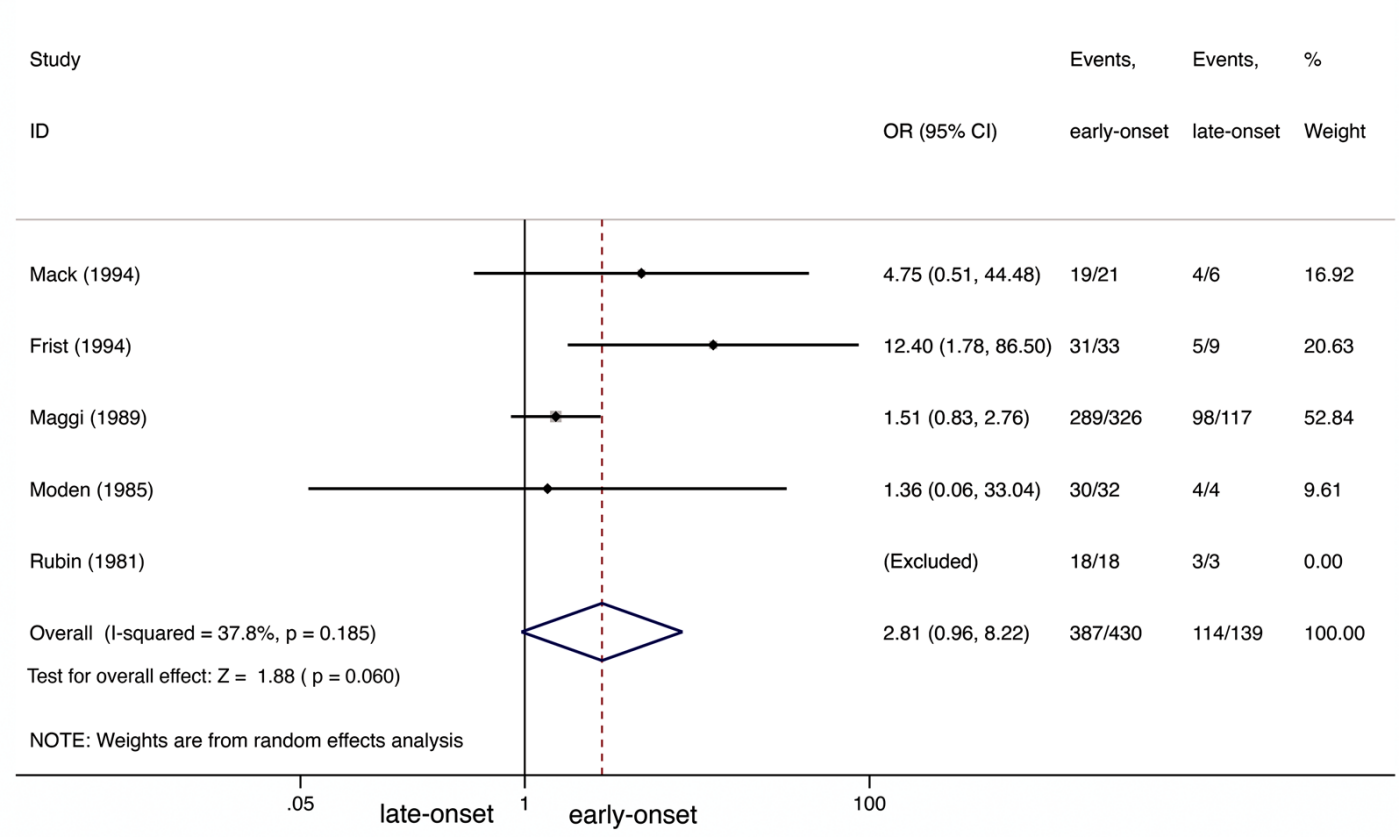
CSR+improvement comparison between early-onset and late-onset NTMG after thymectomy

We performed a further analysis on CSR+ improvement between early- and late-onset NTMG patients, observing no significant difference between the groups (OR 2.81; 95% CI 0.968.22; *I*^2^=37.8%; Fig.4). No evidence of publication bias was detected by Peters test (*P*=0.346).

### Thymectomy versus conservative treatment in late-onset NTMG (meta-analysis)

**Fig. 5:**
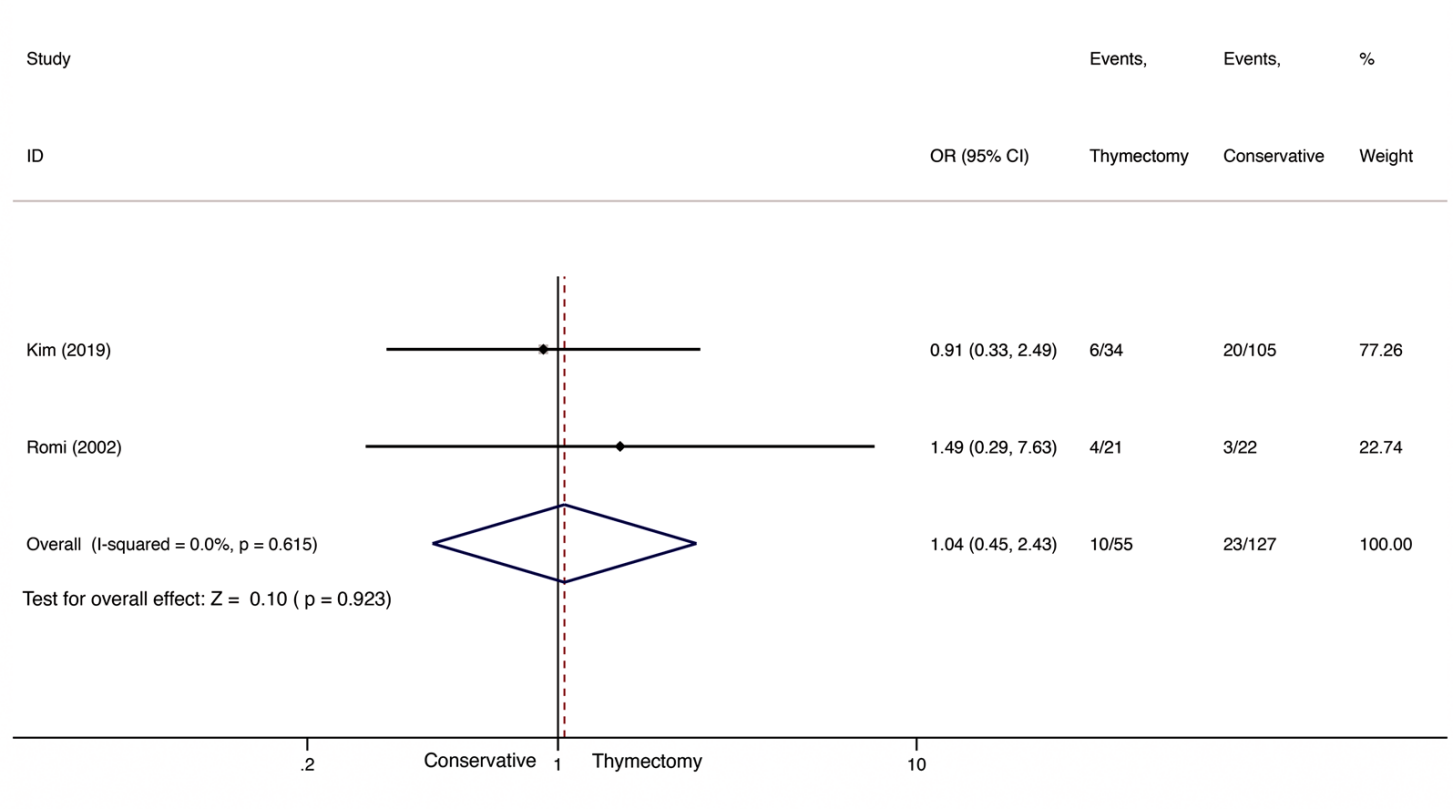
CSR comparison between thymectomy and conservative treatment in late-onset NTMG

For CSR comparison, due to limited data on thymectomy *versus* conservative treatment in late-onset NTMG patients, only two studies, including a total of 55 patients in the thymectomy groups and 127 in the conservative-treatment groups, met our criteria [11, 15]. Meta-analysis results did not reveal any difference between thymectomy and conservative treatment for late-onset NTMG (OR 1.04; 95% CI 0.452.43; *I*^2^=0.0%; Fig.5). Publication bias was not assessed due to the limited number of studies available.

**Fig. 6:**
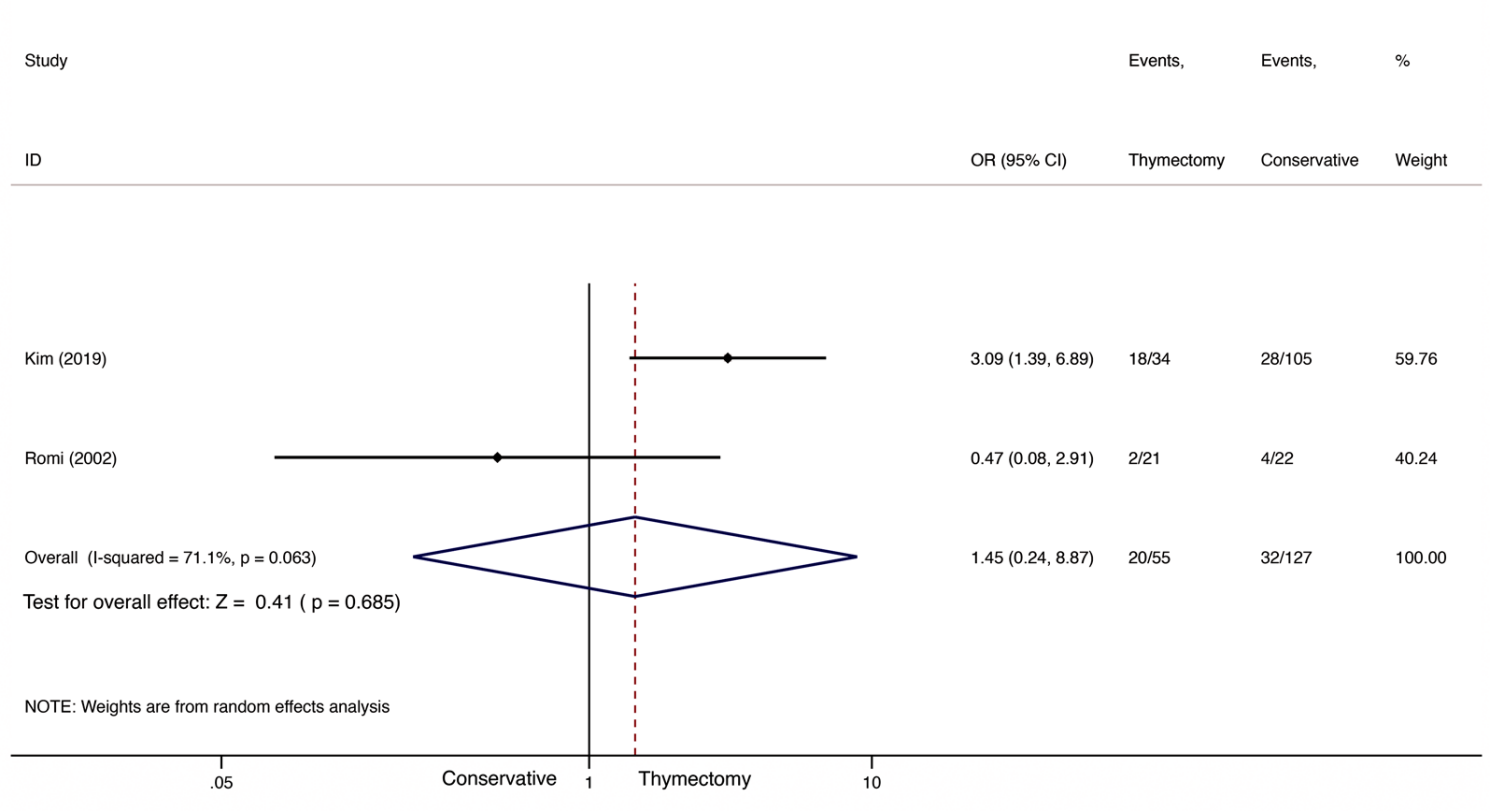
PR comparison between thymectomy and conservative treatment in late-onset NTMG

Similarly, only two studies included PR data on thymectomy *versus* conservative treatment in NTMG patients [11, 15]. We observed no statistically significant difference between the two types of treatment (OR 1.45; 95% CI 0.248.87; *I*^2^=71.1%; Fig.6). Publication bias was, again, not assessed.

**Fig. 7:**
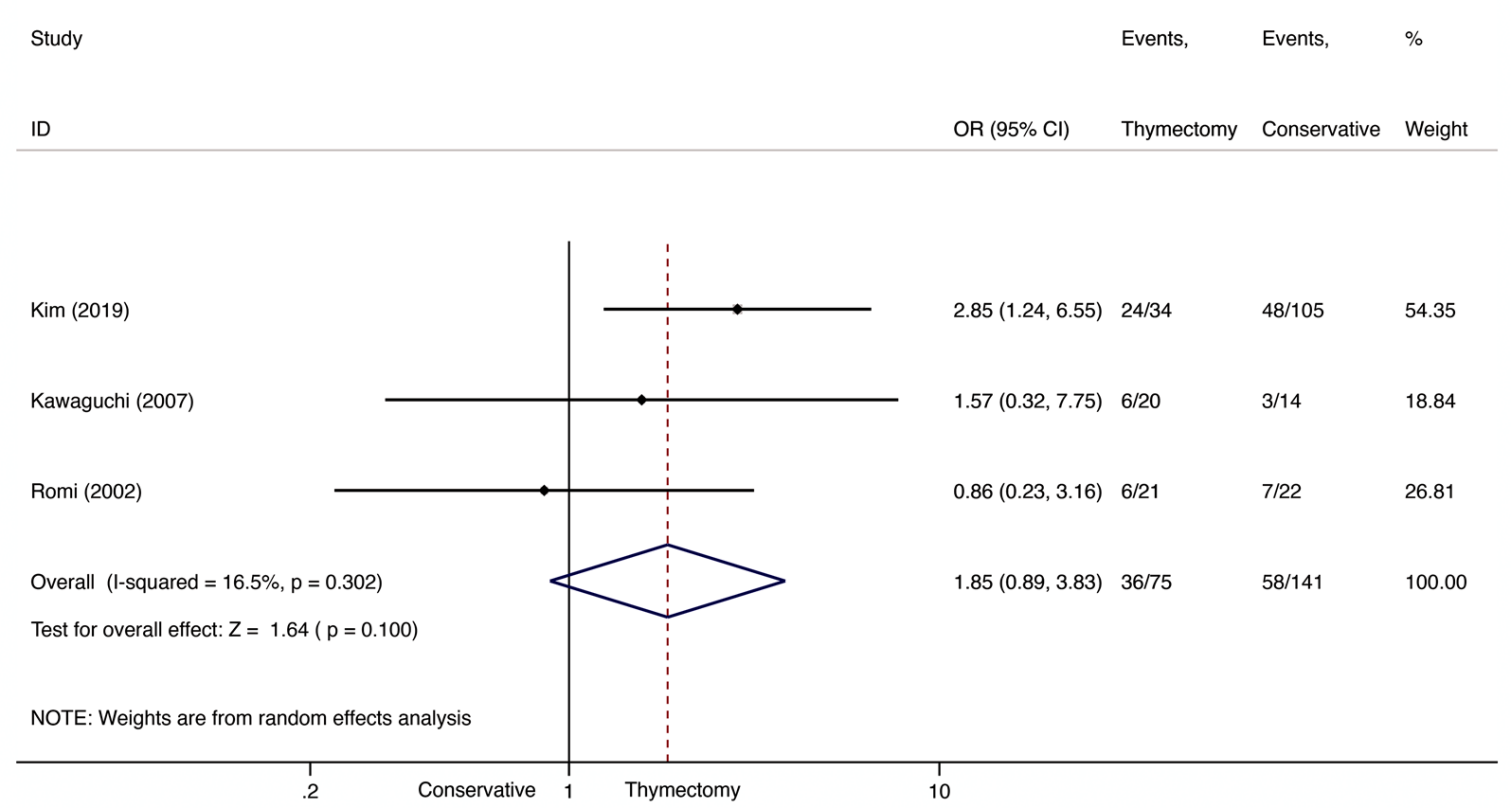
CSR+PR comparison between thymectomy and conservative treatment in late-onset NTMG

A total of three studies [11, 13, 15], including a total of 75 patients in the thymectomy groups and 141 in the conservative-treatment groups, were included for CSR+PR comparison. CSR/PR did not differ significantly between groups (OR 1.85; 95% CI 0.893.83; *I*^2^=16.5%; Fig.7). However, the thymectomy groups achieved CSR/PR at a higher rate than the conservative-treatment groups. We found no evidence of publication bias using Peters test (*P*=0.355).

## Discussion

So far, no consensus has been reached on the appropriate cutoff age to divide early-onset from late-onset myasthenia gravis [5, 6, 30]. Many studies have indicated age as one of the factors associated with MG prognosis after thymectomy. However, most of these studies combined thymomatous and non-thymomatous patients in their statistical considerations, potentially confounding the final results. Our work specifically targeted NTMG patients; we extracted nine studies that compared early- with late-onset NTMG after thymectomy. All of the included studies set a cutoff age between 40 and 50years. We excluded three studies with cutoff ages of<40years. Of the included studies, three found that early age of onset correlated significantly with better prognosis in NTMG after thymectomy [12, 22, 25], while another three studies found no difference [20, 21, 26]. Furthermore, we found that early-onset patients had a 1.95 greater CSR rate than late-onset patients. No difference was seen in improvement or CSR+ improvement rates.

While the benefits of thymectomy in NTMG patients have not been conclusively established, there is increasing evidence to support the positive effects thereof compared with medical treatment [31, 32]. Moreover, using high doses of prednisone and/or immunosuppressants has raised some concerns among neurologists. The MGTX study provided cogent evidence that by undergoing thymectomy, generalized NTMG patients who are AChR-ab^+^ have a 67% probability of achieving optimal control of their disease within 12months. They also benefit from significantly reduced exposure to steroids and immunosuppressants and a much lower rate of treatment-associated symptoms [10]. A meta-analysis by Cataneo et al*.* also shows the benefits of surgical treatment [33]. However, the MGTX trial did not establish the role of thymectomy in late-onset NTMG patients. Based on existing evidence, a recent International Consensus Guidance (ICG) for MG recommends, In non-thymomatous, generalized MG patients with AChR-ab, aged 1850years, thymectomy should be considered early in the disease to improve clinical outcomes and to minimize immunotherapy requirements and need for hospitalizations for disease exacerbations [34].

For NTMG patients age50years, our database searches retrieved only a few studies that directly compared thymectomy versus conservative treatment in late-onset NTMG. The latest cohort study was a landmark analysis by Kim et al*.*, who found that thymectomy patients had a 2.22-fold better chance of achieving PR than medical-treatment patients after adjusting for age, sex, and disease severity [15]. Kawaguchi et al*.* [13] conducted a retrospective study with a relatively small number of patients and found no difference in CSR/PR between the thymectomized and conservative groups. However, subgroup analyses including 22 patients with mild generalized weakness showed that the thymectomized group had a higher rate of CR and a lower frequency of generalized symptoms than the non-thymectomized group at the end of follow-up, indicating that thymectomy is a potentially effective treatment for NTMG patients with mild generalized MG. Due to selection bias, we could not properly synthesize this subgroup data. However, the study by Romi et al*.* [11] showed no significant benefit from thymectomy in late-onset NTMG.

Romi et al*.*s [11] study also examined whether the therapeutic effect of thymectomy was linked to the presence of non-AChR muscular antibodies. CR or PR was seen in 6 of 11 titin antibody-negative but 0 of the 10 titin antibody-positive thymectomized patients; the non-thymectomized cases showed the opposite trend. A limited number of studies have performed similar analyses. Most researchers have focused on preoperative anti-AChR-ab titers. Among our included studies, three evaluated anti-AChR-ab influence CSR in NTMG patients [12, 20, 22]. None of them found evidence to support a correlation between prognosis and these antibodies.

Late-onset MG is often associated with thymic atrophy [7]. Uzawa et al*.* [35] retrospectively reviewed 2-year post-thymectomy prognoses in 39 consecutive generalized NTMG patients who were AchR-ab^+^ (age at onset,>50years). Late-onset NTMG patients with thymic hyperplasia showed a higher rate of remission and received lower prednisolone doses versus patients with involuted thymuses. However, Nakahara et al*.* compared clinical features and postoperative prognoses among three different thymic histologies and found that the clinical course of MG patients with atrophic thymus after thymectomy was superior to those with hyperplasia or thymoma [36]. Whether thymic histology correlates with response to thymectomy in NTMG is still unclear, but we found significant proportional heterogeneity of thymic histology in our three included studies (Table 2). Histological examination showed that all patients in Romis study had thymic atrophy [11]. However, Kawaguchis study [13] provided no thymic-histology data.

**Table 2:** Demographic data of studies comparing thymectomy with conservative treatment in late-onset NTMG

Author/year/coconut	Study design	Study period	Follow-up (y) (mean/range)	Age (y) (cutoff/range)	Thymectomy (events/all)		Conservative (events/all)		Thymic histology hyperplasia/involution/normal	Anti-AChR-ab(+/)	Preoperative classification	Surgical procedures	Medical treatment	NOS score
					CSR	PR	CSR	PR						
Kim/2019/South Korea [15]	Single-center retrospective	19902018	NA/2.511	45 (onset) 50 (thy)*/47.866	6/34	18/34	20/105	28/105	18/16/0	139/0	II 57/III 52/IV 8/V 22 MGFA	NA	Anticholinesterase; corticosteroid; immuno-suppressant	9
Romi/2002/Norway [11]	Single-center retrospective	19691999	NA/25	50/NA	4/21	2/21	3/22	4/22	0/21/0	43/0	Mean 2.9 for thymectomy,2.6 for conservative group. Osserman	TS	Corticosteroid; immuno-suppressant	9
Kawaguchi/2007/Japan (13)	Multicenter retrospective	19992000	9.6/NA	50/5078	6/20		3/14		NA	29/5	Mean 2 for thymectomy,2.5 for conservative group. MGFA	NA	Anticholinesterase; corticosteroid; immuno-suppressant	8

*NTMG* non-thymomatous myasthenia gravis, *AntiAChR-ab* anti-acetylcholine receptor antibody, *CSR* complete stable remission, *PR* pharmacological remission, *TS* trans-sternal thymectomy, *MGFA* Myasthenia Gravis Foundation of America, *NA* not available, *NOS* NewcastleOttawa scale

*Onset of MG at45years of age/when patient underwent thymectomy at50years of age

Considering how surgical procedures correlate with the effects of surgery in NTMG. Liu et al*.* [20] compared right-sided (unilateral) with bilateral VATET and found no difference in long-term outcome. Lin et al*.* [12] compared the results of VATS thymectomy with those of trans-sternal thymectomy and found that the two methods had equivalent CSR rates but the former resulted in shorter hospital stay, less tissue injury, and better cosmetic outcome. In Zielinski et al*.*s [21] comparison of CR rates between basic trans-sternal thymectomy and extended trans-sternal thymectomy, outcomes were considerably better in the extended-thymectomy group. Considering the wide acceptance of extended thymectomy as the current standard procedure [27], we extracted data only from the extended subgroup. Minimally invasive techniques seemed to achieve the same benefits as trans-sternal methods; however, well-designed controlled studies are still needed [28]. Among our included studies, one included a group of patients who received transcervical surgery [25], which can weaken postoperative outcome [28]. Moreover, two studies did not describe the exact surgical procedure [13, 15]. All three studies therefore presented sources of potential heterogeneity in our analyses.

Preoperative severity may correlate with prognosis for thymectomy in NTMG [24, 25]. Of the studies on thymectomy versus conservative treatment in late-onset NTMG that we selected, two found no significant difference in terms of preoperative MGFA classification [11, 15]. One study had more severe patients in its thymectomy group than in its nonthymectomy group [13], although final outcomes were not significantly different between the two groups.

To our knowledge, this is the first systematic review and meta-analysis of thymectomy in late-onset NTMG. Despite the small number of observational studies included, our meta-analysis showed a difference between early- and late-onset NTMG patients in terms of CR but not in the effects of thymectomy versus conservative treatment in late-onset NTMG. However, our study had several limitations. First, all studies included were observational, and such studies should be interpreted with caution. Second, the number of studies included was relatively small. Third, the inherent heterogeneity of patient populations and intervention groups could have introduced unknown sources of bias into the results. More multi-center studies with large sample sizes and well-controlled cases are needed to limit bias in the future. Moreover, the appropriate cutoff age dividing early- from late-onset MG needs to be further investigated in order to develop tailored therapeutic strategies.

## Conclusion

We observed that late-onset NTMG patients had a lower chance of achieving CSR after thymectomy than early-onset patients, but no difference was seen in improvement or in CSR+ improvement rates. Moreover, late-onset NTMG patients did not obtain any benefits from thymectomy *versus* conservative treatments. Thymectomy in late-onset NTMG patients should therefore be performed with caution, and further investigation into cutoff ages is needed to deliver specific therapeutic strategies.

## Data Availability

We searched the PubMed, EMBASE, and Cochrane Library electronic databases.
